# News and Notes

**Published:** 1906-01

**Authors:** 


### NEWS AND NOTES.

Dr. J. W. Cooper, of West Point, Ga., died in Atlanta December 4th.

Joseph H. Hines, M.D., Atlanta, Ga., was married to Miss Cecelia Lopez, of Charleston, S. C., November 15th.

Since January 1 there have been 60,000 cases of typhoid in New York, and 500 deaths in Greater New York alone.

The Cordele Sanatorium will be opened about January 1st by Drs. Thomas J. McArthur and Walter E. Edwards, Cordele.

On November 17, $90,000 was pledged in thirty-five minutes for the building of the Baptist Memorial Sanatorium, Dallas.

Dr. W. S. Goldsmith attended the recent meeting of the Southern Surgical and Gynecological Society in Louisville, Ky.

Dr. C. L. Summey died at his home in Stone Mountain December 9th, after a long illness. Dr. Summey's wife died just two weeks previously.

The trustees of the Presbyterian Hospital, Atlanta, have recommended that the hospital association build a steel fireproof hospital to cost $50,000.

According to press reports Dr. Charles Hicks, a well-known physician of Dublin, Ga., was stricken with partial paralysis while in his office December 16th.

THE *Pall Mall Gazette* says that a great epidemic of imaginary consumption has prevailed in Paris since the close of the recent tuberculosis congress in that city.

---

IN 1895 the degree of doctor of medicine was conferred on 1,011 medical students in the universities of Austria and Hungary. In 1904 only 561 similar degrees were conferred.

---

DR. WM. H. WATHEN, of Louisville, for many years referee for the Kentucky State Board of Health, has resigned, and Dr. Wm. Bailey, of Louisville, has been appointed in his stead.

---

A THREE-STORY brick building occupied in part by the *Alkaloidal Clinic*, at Ravenswood, Ill., with the presses and publishing equipment, was completely destroyed by fire last month. The loss sustained was $200,000.

---

THE administrators of Tulane University have asked the faculty of the medical department of that institution for an expression of opinion regarding the establishment of a school of tropical medicine in connection therewith.

---

DR. W. L. M. COPLIN, Professor of Pathology and Bacteriology in the Jefferson Medical College, has been chosen to succeed Dr. Edward Martin as Director of the Department of Public Health and Charities of Philadelphia.

---

A MAN was recently operated upon in St. Luke's Hospital, New York, for recurrent appendicitis, when there was found in the appendix a 22-caliber bullet. The patient had shot himself accidentally thirteen years before, but the bullet had caused him no inconvenience until recently.

---

THE Newton and Rockdale (Ga.) Counties Medical Society has been organized by Dr. E. C. Davis, of Atlanta, censor for the Fifth

Congressional District. Dr. E. W. Ragsdale, of Starrsville, was elected president, and Dr. W. D. Travis, of Covington, secretary. The meetings of the society are to be held alternately at Covington and Conyers.

---

THE Prussian medical chambers (Aerztekammer) have been discussing the question of the fee to charge when a physician is consulted over the telephone. The medical chamber in Pomerania has announced that the fee is the same for medical advice given over the telephone as when the patient comes to the physician's office. The other organizations will probably adopt the same rule.—*Jour. A. M. A.*

---

A MEETING of the faculty of the Medical College of Virginia was held at Richmond on November 10, 1905, to discuss the broadening and strengthening of the faculty and courses of study with a view of entering into the amalgamation with the University of Virginia. The plans of the faculty are of the most advanced nature in the direction of clinical teaching and post-graduate work. A sub-committee was appointed to draft the details.

---

THE Tri-State Medical Association of Arkansas, Mississippi and Tennessee held its twenty-second annual meeting at Memphis on November 21, 22 and 23, 1905. Officers for the ensuing year were elected as follows: President, Dr. Allen E. Cox, of Helena, Ark.; vice-presidents, Arkansas, Dr. Dickson, of Paragould; Mississippi, Dr. E. J. Johnson, of Yazoo City; Tennessee, Dr. F. J. Runyan, of Clarksville; secretary, Dr. Richmond McKinney, of Memphis; treasurer, Dr. Marcus Haase, of Memphis.

---

AT a meeting held at Savannah on Wednesday, November 8th, The Chatham County (Ga.) Medical Society, an organization, under the auspices of the Georgia State Medical Association, was effected. Officers were elected as follows: President, Dr. J. W. Daniel; vice-president, Dr. Craig Barrow; secretary, Dr. Herman W. Hesse; treasurer, Dr. W. E. Norton. The by-laws provide that officers shall be elected in December. By vote it was determined that the officers chosen should serve until December, 1906.

---

AT a meeting held at Cordele on November 3, 1905, an organization was formed under the auspices of the Georgia Medical Association, and designated as The South Georgia Medical Association. It is composed of physicians from Crisp county and adjacent counties. The following officers were elected: President, Dr. J. T. Gammage, of Pineview, Wilcox county; vice-president, Dr. Archie Griffin, of Luke, Turner county; secretary and treasurer, Dr. T. J. McArthur, of Cordele, Crisp county; board of censors, Dr. V. O. Hubbard, of Arabi; Dr. J. A. Ward, of Cordele; and Dr. L. A. Williams, of Abbeville.

---

DEATH FROM A WASP STING.—The following remarkable case was investigated by the Sussex coroner. The wife of a workingman was drinking a glass of stout when she suddenly felt a pain in the throat as a result of some body which she had imbibed with the liquid. She coughed it out and found that it was a wasp. Beyond the natural fright and some discomfort in the throat, she seemed but little the worse for her experience, and for the next few days was comparatively well, so that medical advice was not sought. Then she felt faint and ill, but continued to pursue her ordinary vocation until the sixth day after the sting, when she called in a physician. He found little evidence of serious illness, but her symptoms rapidly increased and assumed a grave character. Cellulitis of the throat and nasal passages developed and death from septicemia followed in two days. This case differs from the ordinary cases in which dangerous symptoms follow wasp stings in that an interval elapsed between the sting and the onset of symptoms, and that these were due to cellulitis, and not to toxemia.—*London letter Jour. A. M. A., December 2, 1905.*

